# Differential Activity and Expression of Proteasome in Seminiferous Epithelium During Mouse Spermatogenesis

**DOI:** 10.3390/ijms26020494

**Published:** 2025-01-09

**Authors:** Héctor Zapata-Carmona, Emilce Silvina Díaz, Patricio Morales, Marco Jara

**Affiliations:** 1 Laboratorio de Biología de la Reproducción, Departamento Biomédico, Facultad de Ciencias de la Salud, Universidad de Antofagasta, Antofagasta 1240000, Chile; hector.zapata@uantof.cl (H.Z.-C.); emilce.diaz@uantof.cl (E.S.D.); patricio.morales@uantof.cl (P.M.); 2 Instituto Antofagasta, Universidad de Antofagasta, Antofagasta 1240000, Chile

**Keywords:** spermatogenesis, cycle of the seminiferous epithelium, proteasome, proteasome assembly, proteolysis, proteasome regulatory complex

## Abstract

Proteasome-mediated protein degradation is essential for maintaining cellular homeostasis, particularly during spermatogenesis, where extensive cellular transformations, such as spermatid differentiation, require precise protein turnover. A key player in this process is the ubiquitin–proteasome system (UPS). This study aimed to investigate proteasome enzymatic activity at different stages of the spermatogenic cycle within the seminiferous tubules of mice and explore the regulatory mechanisms that influence its proteolytic function. Specifically, we assessed the trypsin-like, chymotrypsin-like, and peptidyl-glutamyl-peptide-hydrolyzing (PGPH) activities of the proteasome. Additionally, we examined the expression of catalytic and structural subunits of the 20S core, the assembly of the 20S core with regulatory complexes, and the phosphorylation status of proteasome subunits in various segments of the seminiferous tubules. Our findings demonstrated distinct patterns of proteasomal enzymatic activity in the analyzed segments. While the expression levels of structural and catalytic subunits of the 20S core remained consistent, significant differences were detected in the assembly of the 20S core, the expression of regulatory complexes, and the phosphorylation of proteasome subunits mediated by protein kinase A. These results indicate that proteasomal activity is finely regulated through multiple mechanisms depending on the specific stage of the seminiferous epithelial cycle, highlighting the complexity of proteostasis during spermatogenesis.

## 1. Introduction

Spermatogenesis is a complex process that occurs in the seminiferous tubules, where spermatogonia undergo morphological and biochemical transformations to become specialized cells [1,2]. In numerous mammalian species, germ cells are organized into stages of the seminiferous epithelium cycle, identifiable by their distinct morphology in histological cross-sections [3,4]. The functionality of Sertoli cells varies depending on the type of germ cell with which they are associated in each segment of the seminiferous epithelium. This observation indicates that the cellular processes occurring within each stage of the seminiferous epithelium are distinct and characteristic of a specific cellular grouping [5].

In adult mouse testes, germ cells form 12 distinct stages (I–XII), each with a specific cellular composition [6,7]. These stages, arranged along the seminiferous tubules, span approximately 8.6 days, with full spermatogenesis taking 33 days [3,8]. Histological sections of mouse testes can be classified into specific stages based on the germ cells present around the tubule lumen [9]. Throughout spermatogenesis, particularly during spermatid differentiation, extensive cellular remodeling occurs, predominantly involving the ubiquitin–proteasome system (UPS) [10]. The UPS is responsible for the degradation of the majority of short-lived normal, defective, or misfolded intracellular proteins [11]. In this system, substrates are typically designated for degradation via covalent attachment to multiple ubiquitin molecules. Ubiquitin, a monomeric protein comprising 76 amino acid residues with a molecular weight of 8.5 kDa [12,13], functions as the central component of the UPS. Ubiquitination, a post-translational modification, is executed by a large array of proteins. Currently, three enzymes responsible for ubiquitination have been identified—E1 (activating enzyme), E2 (conjugating enzyme), and E3 (ligase enzyme)—which operate sequentially to facilitate the binding of multiple ubiquitin molecules to the lysine residues of proteins designated for degradation or recycling [14]. Once tagged with polyubiquitin chains, proteins are rapidly degraded by the proteasome.

The proteasome is a multi-enzyme complex that exhibits proteolytic activity and comprises multiple subunits [10,15]. It consists of a 20S core that is associated with a regulatory complex. The 20S core is structured with two pairs of homologous rings, each containing seven subunits. The two outer rings consist of α-type subunits (PSMA1-7) [16], whereas the two inner rings are formed by β-subunits (PSMB1-7). Among these, β1 (PSMB6), β2 (PSMB7), and β5 (PSMB5) are catalytically active and possess caspase-like (or peptidyl-glutamyl-peptide-hydrolyzing (PGPH)), trypsin-like, and chymotrypsin-like activities, respectively [10,17]. Proteasome activity is intricately modulated by regulatory complexes that bind to α-rings. In the absence of these regulatory complexes, the proteasome channel remains closed to the N-terminus of seven different units, thereby repressing proteolytic activity [18,19]. Four regulatory complexes that influence proteasomal activity have been identified in mammalian cells, namely, 19S, PA28α/β, PA28γ, and PA200.

The archetype of the eukaryotic proteasome is the 26S proteasome, which consists of a 20S core and two 19S regulatory complexes [20]. PA200 is highly expressed in the testis and is predominantly localized within the nucleus, suggesting its potential involvement in the degradation of proteins implicated in post-crossing DNA repair processes [21,22,23]. Studies involving knockout mice lacking PA200 have revealed a significant decrease in fertility attributed to impaired spermatogenesis, highlighting the critical role of PA200 in this process [24].

In mice, several enzymes involved in ubiquitination are highly expressed in the testes and appear to be involved in spermatogenesis. Specifically, HERC4, an E3 ligase enzyme, has been linked to sperm maturation for ensuring normal fertility [25,26]. UBC4, an E2 enzyme specific to the mouse testis, exhibits predominant expression of both its mRNA and protein in round spermatids, suggesting its importance in the early maturation of mouse sperm [27,28,29]. Similarly, the C-terminal ubiquitin hydrolase, responsible for protein deubiquitination, is expressed in mouse testes [30] and plays a fundamental role in spermatogenesis, contributing both to the mitotic proliferation of spermatogonial stem cells and the meiotic differentiation of spermatocytes into spermatids [31,32,33].

In mammals, the UPS actively participates in the later stages of spermatogenesis, notably in chromatin remodeling, particularly in the replacement of histones by transition proteins and subsequently by protamines [10,34,35,36]. Mouse histone H2A is ubiquitinated and discarded in the cytoplasmic droplet of sperm before its release from the seminiferous epithelium [37].

The UPS is the primary system for cytosolic protein degradation in eukaryotic cells. The UPS plays a critical role in this process, given the extensive degradation of proteins and organelles during spermatogenesis [10,38]. However, there is limited information on proteasome activity at the different stages of spermatogenesis. Most studies have focused on identifying UPS components at the testicular level [10,38,39].

To date, no studies have assessed proteasome activity across various stages of the mouse seminiferous epithelium, nor have they investigated potential mechanisms regulating the enzymatic activity of the complex. Therefore, the objective of this study was to characterize the enzymatic activity of the proteasome in different segments of the mouse seminiferous epithelium and to explore the potential mechanisms regulating its proteolytic activity.

## 2. Results

**Figure 1:**
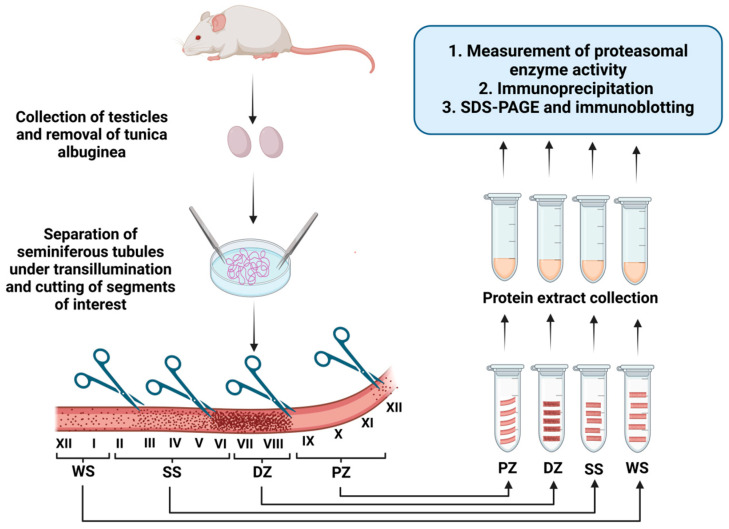
A schematic of the experimental design. Testes from sexually mature adult mice were obtained via abdominal incision and decapsulated to remove the tunica albuginea. The seminiferous tubules were mechanically separated from the interstitial tissue using fine forceps under a transillumination dissecting microscope. Through transillumination, different segments of interest corresponding to the WS (stages XII–I), SS (stages II–VI), DZ (stages VII and VII), and PZ (stages IX–XI) zones were identified. Sperm released into the lumen were flushed out of the lumen by washing. These segments were sonicated, and the supernatant from the homogenate was collected as the protein extract used for this study. (Created by Zapata-Carmona et al., via BioRender, https://app.biorender.com/, on 29 November 2024).

In the present study, a detailed analysis of proteasome activity was conducted in different segments of the seminiferous tubules of mice. The 12 stages of the seminiferous epithelium cycle were grouped into four zones using the nomenclature described for this technique [40,41], allowing for the precise evaluation of changes in proteasomal proteolytic activity and its potential regulatory mechanisms throughout the spermatogenic cycle. The experimental design is illustrated in Figure 1, which provides an overview of the sampling and analysis processes. Based on this approach, the following sections present the results of each evaluation.

### 2.1. Proteasome Activity in Different Zones of the Mouse Seminiferous Epithelium

**Figure 2:**
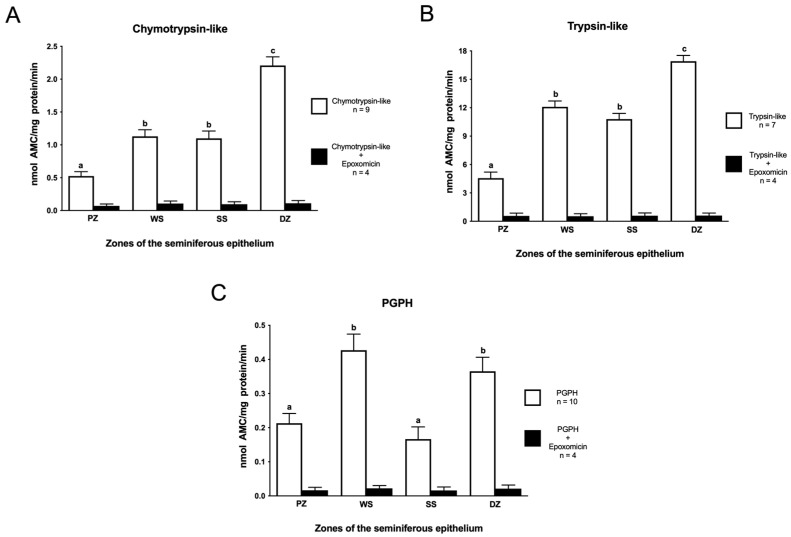
Proteasomal activity in mouse seminiferous epithelium. (**A**) The chymotrypsin-like, (**B**) trypsin-like, and (**C**) PGPH activities were evaluated in protein extracts from the PZ (stages IX–XI), WS (stages XII and I), SS (stages II–VI), and DZ (stages VII and VIII) zones, corresponding to the different zones of the mouse seminiferous epithelium. Protein extracts were incubated with the appropriate fluorogenic substrates in the presence (black bars) or absence (white bars) of 10 µM epoxomicin. Data are expressed as mean ± SEM of the replicates indicated in each activity. Different letters indicate statistically significant differences (*p* < 0.05) between the groups.

The highest proteasome chymotrypsin-like activity was observed in the DZ, which contains stages VII and VIII (Figure 2A, *p* < 0.05), and the lowest activity was observed in the PZ, which contains stages IX, X, and XI. There were no significant differences between the WS and SS zones. Similar results were observed for trypsin-like activity (Figure 2B), which was greater in the DZ and lower in the PZ (*p* < 0.05). Behavior in terms of PGPH-like activity was somewhat different from that for the other two activities (Figure 2C). The WS and DZ zones showed the highest PGPH-like activity compared to the PZ and SS zones (*p* < 0.05). To confirm that the peptides used were specific to the three proteasome activities, each experiment was conducted in the presence of the specific proteasome inhibitor epoxomicin (Figure 2A–C). The results show that all three proteasome activities decreased considerably in the four zones studied, confirming that these measured activities were specific to the proteasome. Note that specific trypsin-like activity was 10 and 40 times higher than the specific chymotrypsin-like and PGPH-like activities, respectively.

### 2.2. Protein Levels and Assembly of Proteasome Subunits in Different Zones of the Mouse Seminiferous Epithelium

**Figure 3:**
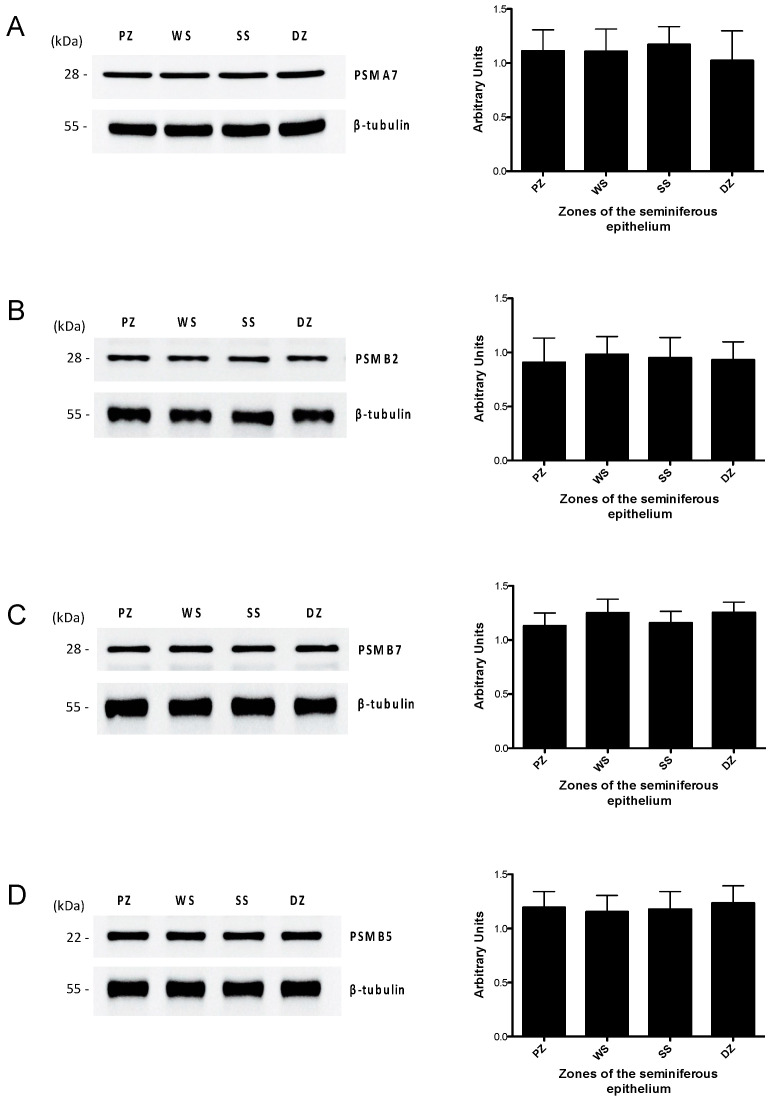
Protein level of the 20S proteasome subunits in the mouse seminiferous epithelium. The protein levels of the structural subunits PSMA7 (**A**) and PSMB2 (**B**), and the catalytic subunits PSMB7 (**C**) and PSMB5 (**D**), were determined by Western blot in the PZ (stages IX–XI), WS (stages XII and I), SS (stages II–VI), and DZ (stages VII and VIII) zones. For densitometric analysis, β-tubulin load was used as a control. The bars represent the mean ± S.E.M. from three different experiments.

Western blotting results indicate that the protein levels of two proteasome structural subunits, PSMA7 (Figure 3A) and PSMB2 (Figure 3B), did not show differences in the different zones under study. The PSMA7 and PSMB2 proteins were detected in the four study areas, with a molecular weight of 28 kDa. We also evaluated the protein levels of the two proteasome catalytic subunits (Figure 3C,D). A 28 kDa molecular weight band corresponding to the PSMB7 protein with trypsin-like activity and a 22 kDa molecular weight band corresponding to the PSMB5 protein with chymotrypsin-like activity were observed in the four study areas. In agreement with the results for the expression of the structural subunits, the catalytic subunits did not present significant variation in protein expression between the study areas.

These results indicate that although the three enzymatic activities of the proteasome differ in the zones of the seminiferous tubule, there are no differences in the protein levels of the structural or catalytic subunits. This suggests that the differences in proteasomal activities are due to the specific regulation of proteasome function during the development of the mouse germline. Since none of the areas studied showed changes in the total levels of the proteasome subunits (catalytic and regulatory), there could be differences in proteasomal assembly.

**Figure 4:**
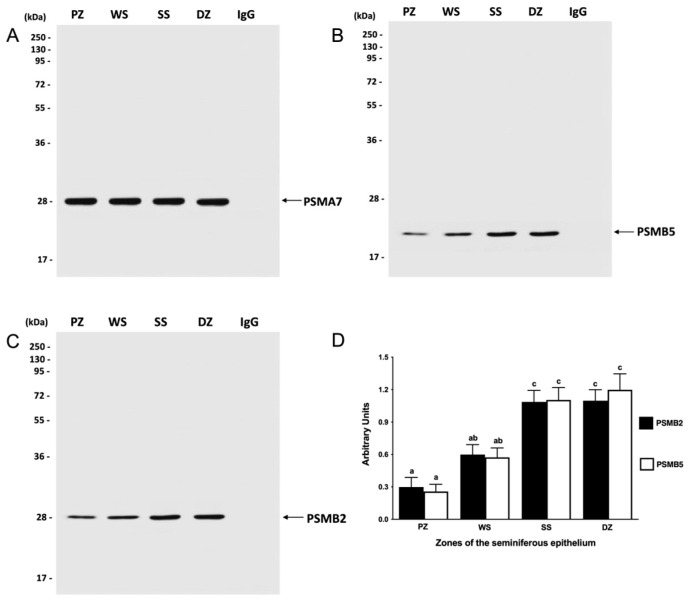
Assembly of the 20S proteasome in the mouse seminiferous epithelium. Extracts from the PZ (stages IX–XI), WS (stages XII and II), SS (stages II–VI), and DZ zones (stages VII and VIII) were immunoprecipitated with an anti-proteasome antibody and were detected by Western blot using an antibody against the PSMA7 subunit (**A**), the PSMB5 subunit (**B**), and the PSMB2 subunit (**C**). For densitometric analysis, the PSMA7 subunit was used as a control (**D**). The bars represent the mean ± S.E.M. from three different experiments. Different letters indicate significant statistical differences at *p* < 0.05.

Thus, the next experiment was designed to evaluate the degree of assembly of this complex in the different zones of the seminiferous epithelium. In each study zone, proteasomes were immunoprecipitated using an anti-PSMA7 proteasomal subunit antibody. This antibody immunoprecipitated both PSMA7, which is part of the 20S proteasome (assembled), and free PSMA7 (not assembled), allowing us to evaluate the total number of PSMA7 subunits present in the extracts. As a control for the immunoprecipitation protocol, the precipitated proteins were tested by Western blotting using an antibody against the PSMA7 subunit (Figure 4A). Similar amounts of PSMA7 subunits were observed in each segment. As an indication of the expression of the assembled 20S complex, we then evaluated the presence of other proteasome subunits in the same sample. The presence of other proteasome subunits associated with the PSMA7 subunit indicated that they were co-immunoprecipitated with the PSMA7 subunit, which is part of the 20S core. Using the same PVDF membrane, we evaluated the presence of the PSMB5 subunit, which corresponds to the catalytic subunit of the 20S proteasome with chymotrypsin-like activity. The PSMB5 protein was detected in the four study areas, with a molecular weight of 22 kDa (Figure 4B). A greater band intensity was observed in the SS (stages II–VI) and DZ (stages VII and VIII) zones than in the other zones. To corroborate the previous results, the assembly was evaluated in the presence of the PSMB2 subunit, which corresponds to a structural subunit of the 20S proteasome. The PSMB2 protein was detected in the four study areas with a molecular weight of 28 kDa (Figure 4C). In agreement with the results observed for the PSMB5 subunit, the PSMB2 subunit was more abundant in the SS and DZ zones than in the other evaluated zones. The densitometric analysis of the PSMB5 and PSMB2 subunits (Figure 4D) indicated that the SS and DZ zones showed significantly greater proteasome assembly than the PZ and WS zones (*p* < 0.05). There were no significant differences between the PZ and WS zones.

### 2.3. Association Between the 20S Core and Regulatory Complexes 19S and PA200 in Different Zones of the Mouse Seminiferous Epithelium

**Figure 5:**
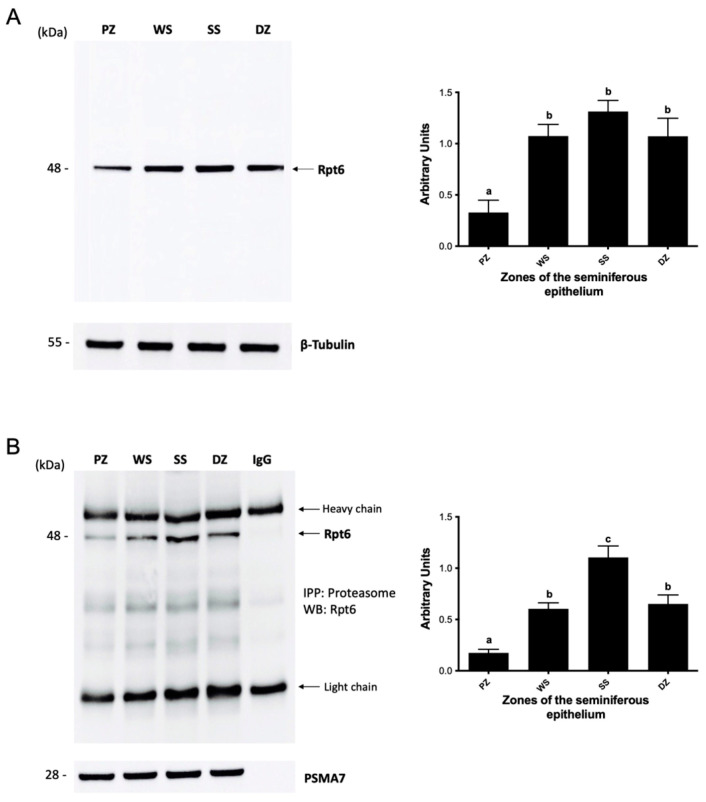
Association between the 20S core and the 19S regulatory complex in the mouse seminiferous epithelium. The protein level of the Rpt6 subunit (**A**) and assembly by immunoprecipitation (**B**) were determined in the extracts from the PZ (stages IX–XI), WS (stages XII and I), SS (stages II–VI), and DZ (stages VII and VIII) zones. Immunoprecipitation of the proteasome was performed with an anti-PSMA7 proteasome subunit antibody. For densitometric analysis, the protein load of β-tubulin (**A**) and the PSMA7 subunit (**B**) was used. The bars represent the mean ± S.E.M. from three different experiments. Different letters indicate significant statistical differences at *p* < 0.05.

Proteasome activity is modulated by regulatory complexes that bind to the 20S core. In this study, we evaluated the association between core 20S and complexes 19S and PA200. The 19S regulatory complex is composed of 18 subunits; the Rpt6 subunit is one of them. We evaluated the presence of the Rpt6 subunit in the protein extracts and proteasome immunoprecipitates from the study zones. The results showed that the Rpt6 subunit was detected in all study areas, with a relative molecular weight of 48 kDa (Figure 5). The protein level of the Rpt6 subunit in the total extracts (Figure 5A) was significantly lower (*p* < 0.05) in the PZ (stages IX, X, and XI) than in the other zones under study. No significant variations in Rpt6 protein levels were observed among the WS, SS, and DZ zones. Immunoprecipitation results, which are indicators of the degree of binding of the Rpt6 protein to the 20S core constituting the 26S proteasome, show that the SS zone (stages II–VI) presents a greater amount of Rpt6 associated with the 20S “core” compared to the other evaluated zones (Figure 5B). This suggests that in the SS zone, the 20S proteasome is mostly assembled with the 19S proteasome. Densitometric analysis indicated that the 26S proteasome in the SS zone was present at a significantly higher level than in the other three zones (*p* < 0.05). The identification of the PSMA7 subunit used to immunoprecipitate the 26S complex, which served as a protein charge control for these experiments, showed that there were no differences in the amount of charged proteins in each lane of the gel (Figure 5B). The lowest amount of the 26S proteasome was detected in the PZ (stages IX, X, and XI). No significant differences were observed between the WS and DZ zones.

**Figure 6:**
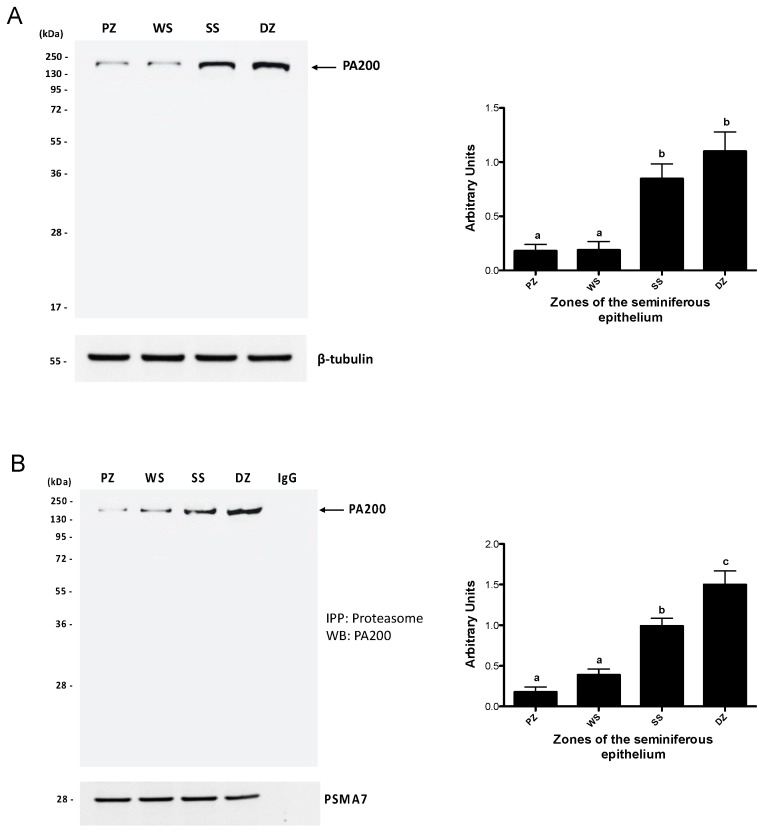
Association between the 20S core and the PA200 regulatory complex in the mouse seminiferous epithelium. The protein levels of PA200 (**A**) and assembly by immunoprecipitation (**B**) were determined in the extracts from the PZ (stages IX–XI), WS (stages XII and I), SS (stages II–VI), and DZ (stages VII and VIII) zones. Immunoprecipitation of the proteasome was performed with an anti-PSMA7 proteasome subunit antibody. For densitometric analysis, the protein load of β-tubulin (**A**) and the PSMA7 subunit (**B**) was used. The bars represent the mean ± S.E.M. from three different experiments. Different letters indicate significant statistical differences at *p* < 0.05.

Considering that the PA200 regulatory complex is highly expressed in the testis and that knockout mice for this complex are infertile, next, we analyzed the protein levels of this complex in the total extract and the assembly of this complex with the 20S proteasome in each of the study zones. The results showed that the PA200 complex with a molecular weight of 200 kDa was detected in all study areas (Figure 6). The protein levels of PA200 in the total extract (Figure 6A) were significantly lower (*p* < 0.05) in the PZ (stages IX, X, and XI) and WS (stages XII and I) zones than in the SS and DZ zones, which presented the highest protein level of this complex. Immunoprecipitation results indicating the degree of assembly of PA200 with the 20S proteasome show that the DZ (stages VII and VIII) exhibited the greatest band density (*p* < 0.05), suggesting that this zone had the highest level of PA200-assembled proteasomes (Figure 6B). The lowest amount of PA200-assembled proteasomes was detected in the PZ (stages IX, X, and XI) and WS (stages XII and I) zones. As can be seen in the figure, the behavior of assembled PA200 is similar to that observed in the measurement of total extracts. However, a significant difference can be observed between the DZ and SS zones (*p* < 0.05).

### 2.4. Phosphorylation of Proteasome Subunits by Protein Kinase A (PKA)

Proteasome subunits undergo post-translational modifications. Among these modifications, phosphorylation is the most prevalent, particularly in 20S core subunits [42]. Studies in other cell models have demonstrated that PKA phosphorylates certain 20S proteasome subunits in vitro, resulting in increased chymotrypsin-like activity and PGPH activity [43]. Consequently, we proceeded to assess the level of PKA-phosphorylated proteasome subunits in each of the zones under investigation. To evaluate this, the proteasome was immunoprecipitated, and the phosphorylated subunits were analyzed by Western blotting using an antibody against phosphorylated PKA substrates.

**Figure 7:**
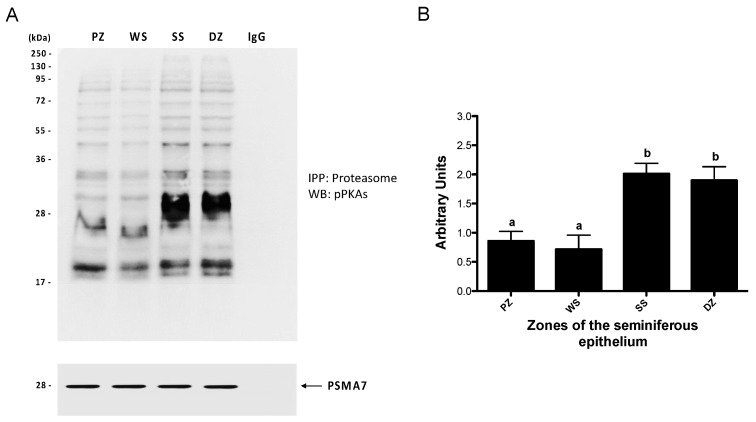
Phosphorylation of proteasome subunits by protein kinase A in mouse seminiferous epithelium. The proteasome was immunoprecipitated with an anti-PSMA7 proteasome subunit antibody in the extracts from the PZ (stages IX–XI), WS (stages XII and I), SS (stages II–VI), and DZ (stages VII and VIII) zones. The phosphorylation of proteasomal subunits by PKA was evaluated by Western blot (**A**) using an antibody against phosphorylated PKA substrates (pPKAs). For densitometric analysis, the PSMA7 subunit was used as a control (**B**). The bars represent the mean ± S.E.M. of the total signal from phosphorylated proteasome components from three different experiments. Different letters indicate statistically significant differences (*p* < 0.05).

The results indicate (Figure 7) that the SS (stages II–VI) and DZ (stages VII and VIII) zones exhibited a significantly higher degree of phosphorylation (*p* < 0.05) compared to the other evaluated zones. A significant difference was not demonstrated between the PZ and WS zones when the level of phosphorylation of their constituent subunits of the 26S proteasome was assessed. These findings suggest that proteasomal subunits are phosphorylated by PKA during spermatogenesis.

## 3. Discussion

In the present study, we measured the three proteasome activities in mouse seminiferous tubule segments at different stages of spermatogenesis. The results obtained indicate that all three proteasome activities are present in all the segments of the seminiferous epithelium analyzed, but the specific activities are different in the different segments. It has been shown before that proteasome activity is essential for the development of a series of cellular events that depend on the degradation of cytosolic proteins [44,45,46]. Many of the cellular events described in somatic cells are carried out during spermatogenesis, along with other events dependent on protein degradation that are absolutely particular to the spermatogenetic process, such as the replacement of histones by protamines [35,36,47,48]. Selective protein degradation, mediated by the proteasome, occurs at particular times in the life cycle of male germline cells. Within the seminiferous tubules, the germline cells form particular associations called the stages of the seminiferous epithelium. In this study, the twelve stages of the mouse seminiferous epithelium cycle were grouped into four zones using the nomenclature described for this technique [40,41]. As expected, we detected the three proteasomal activities in all the seminiferous tubule zones studied. However, the activities were different in the four zones under study. A measurement of these differences in specific proteasome activity during mouse spermatogenesis was not available until now.

The highest specific proteasome activity for the three enzymatic activities analyzed in this investigation was observed in the DZ, corresponding to stages VII and VIII of the mouse seminiferous epithelium cycle, where the final events of spermiogenesis and the release of spermatids to the lumen of the seminiferous epithelium occur [49,50]. During the final phase of spermiogenesis, excess cytoplasm from elongated spermatids is eliminated, forming a residual body. Several UPS components have been identified in the residual bodies of different mammalian species. Specifically, it has been reported that certain UPS components work in conjunction with ALOX15 to facilitate the removal and degradation of cytoplasmic sperm remnants [51]. ALOX15 plays a critical role in sperm maturation, particularly in lipid metabolism and the clearance of residual bodies. Its dysregulation can negatively affect male fertility [52]. In mice, several E3 ubiquitin ligases have been identified during the final phase of spermiogenesis, particularly in the elimination and degradation of histones, which facilitate sperm DNA condensation [38,47,53,54]. During this process, histones within the spermatid are tagged with ubiquitin and subsequently degraded by the proteasome. The E2 ubiquitin-conjugating enzyme UBE2J1 has been shown to be essential for spermiogenesis in mice [29,55]. Sperm from Ube2j1 knockout mice display excess residual cytoplasm around the acrosome, neck, and midpiece of the flagellum [29]. This failure in cytoplasmic removal ultimately leads to male infertility [55]. Additionally, RNF133, an E3 ubiquitin ligase expressed in mouse testes, has recently been identified as critical for sperm function during spermiogenesis [56]. The interaction between RNF133 and UBE2J1 suggests that these proteins may collaborate in this process during spermatogenesis [56]. Knocking out Rnf133 in male mice results in severe subfertility, characterized by abnormal head–neck morphology [56]. As expected, our results showed that the DZ exhibited the highest proteasomal activity. Moreover, Tipler [57], working with isolated cells rather than seminiferous tubules, reported that the highest chymotrypsin-like activity was observed in elongated spermatids, whereas the lowest activity was detected in primary spermatocytes and round spermatids. Our findings also demonstrated that the lowest proteasome activity was observed in stages IX, X, and XI, corresponding to the PZ, where spermatids are in the early phases of spermiogenesis. Intermediate proteasomal activity was observed in the WS and SS zones. This is consistent with the fact that spermatogenesis is a sequential and systematic process that occurs within specific segments of the seminiferous tubule [50,58]. Since spermiogenesis is a continuous and progressive process of cell differentiation, it must be taken into consideration that the more-differentiated spermatids (S16) found in stages VII and VIII originate from the less-differentiated spermatids (S14 and S15) present in stages II to VI of the seminiferous epithelium, which are located in the SS zone [49,50]. Therefore, the number and density of elongated spermatids should not differ significantly between these two zones, suggesting that the observed differences in proteasome activity were not due to variations in spermatid number within the studied segments.

On the other hand, it is well established that mouse spermatozoa possess enzymatic activities [57,59,60]. However, in our study, spermatozoa were absent from the analyzed samples because of their displacement during tubule isolation and washing. Therefore, the enzymatic activity observed in our study originated exclusively from cells present in the seminiferous epithelium. Considering these observations, we propose that the differences observed in this study could be attributed to variations in the physiological processes to which specific cell types are subjected within a given segment of seminiferous tubules.

The application of inhibitors of the proteolytic activity of the proteasome is the primary method for accurately evaluating the functions of the UPS [61,62]. In this study, we utilized the inhibitor epoxomicin. Epoxomicin is a cell-permeable inhibitor that potently, selectively, and irreversibly inhibits all three proteasome activities [63,64]. The results obtained in all the analyzed areas demonstrate that the enzymatic activity observed in each of the study areas corresponds specifically to the proteasome.

The results indicate that proteasome activity varied when comparing the different seminiferous tubule segments analyzed. We postulate that these differences are attributable to a lower expression of the constituent subunits of this enzymatic complex in the different stages of the mouse seminiferous epithelium. Upon evaluating the protein levels of some of the 20S proteasome subunits, we observed that both catalytic and structural subunits were present in similar quantities in the different regions of the mouse seminiferous epithelium. However, a crucial point to consider is that proteasome subunits undergo a cascade of intermediate stages before their assembly to constitute the complete 20S functional proteasome [65]. Therefore, the presence of subunits alone does not directly reflect the activity of the proteasome or the presence of a functional proteasome. Considering that the number of subunits was consistent across the different study areas, we concluded that the difference in activity is more likely due to differential regulation of the proteasome in the different stages of the cycle of the mouse seminiferous epithelium. There is diverse evidence that the activity of the proteasome can be modulated by various mechanisms such as assembly [66,67], the presence of regulatory complexes [68,69,70], and/or post-translational modifications of the proteasome subunits [42,69].

Upon evaluation of the 20S proteasome assembly in various regions of the mouse seminiferous epithelium, it was observed that the highest proportion of assembled proteasome was present in the SS and DZ zones, corresponding to the final stages of spermatogenesis. The lowest level of assembly was detected in the PZ and WS zones. A partial correlation exists between assembly and enzymatic activity, as the highest activity and assembly were observed in the DZ, while the lowest activity and assembly were found in the PZ. The 20S proteasome has been shown to have proteolytic activity in vitro [71]. However, it remains unclear whether the 20S proteasome is biologically active within the cell, possessing intrinsic protease activity, or whether its primary function is to serve as a reserve complex for rapid assembly with regulators when required [68]. The degree of 20S proteasome assembly observed in this study cannot fully elucidate the differences in enzymatic activities across the various zones of the seminiferous epithelium. The SS zone exhibited the same degree of 20S proteasome assembly as the DZ, yet it possessed lower chymotrypsin-like and trypsin-like enzymatic activities. It is evident that additional factors must influence the degree of proteasome activity.

Next, we evaluated the association of the 20S proteasome to regulatory complexes. The Rpt6 protein level, a subunit of the 19S regulatory complex [72,73], was elevated in the total extracts of the WS, SS, and DZ areas from the different study zones. However, immunoprecipitation studies indicated that the SS zone exhibits the highest association of the 19S regulatory complex with the 20S proteasome. This suggests the existence of a prior mechanism that facilitates the formation of this complex to a greater extent in one zone compared to another, considering that equivalent 19S protein levels were observed in all three zones (WS, SS, and DZ). Post-translational modifications of specific subunits of certain regulatory complexes, such as Rpt6 phosphorylation, have been demonstrated to enhance local proteasome activity [74,75,76]. Interestingly, the SS zone does not exhibit the highest proteasome activity, but rather intermediate activity compared to the other study zones. This phenomenon may be attributed to the nature of proteins that require degradation in the stages corresponding to the SS zone. The function of the 19S activator is to participate in the degradation of polyubiquitinated proteins [73,77]. Conversely, the protease activator PA200 promotes degradation independently of ubiquitin [23,77]. PA200 is a regulatory complex that binds to the ends of the 20S particle and is present in all mammalian tissues but is highly expressed in the testis [78]. Upon evaluation of the assembly, it is observed that the DZ exhibits the highest association of the 20S proteasome with the PA200 regulatory complex, which corresponds to the final stages of spermatogenesis. Notably, this observation correlates with the enzymatic activity of the proteasome, where the highest activity level corresponds to this study area. The deletion of PA200 has been reported to significantly reduce the fertility of male mice due to severe defects in spermatogenesis [24]. Qian [77] demonstrated that proteasomes associated with the PA200 regulatory complex in the testis are essential for the degradation of acetylated histones during spermatogenesis. Recent studies have demonstrated that mice deficient in both PA200 and ECPAS (double knockout) exhibit significantly reduced proteasome activity in the testes and epididymides, leading to infertility [79]. Moreover, ultrastructural and microscopic analyses revealed that sperm from these mice show disorganization of the mitochondrial sheath [79], suggesting that PA200 plays an important role in spermiogenesis.

Proteasome subunits can undergo post-translational modifications which may exert regulatory effects on the activity of the proteasome, with the phosphorylation of 20S proteasome subunits being the most prevalent [42,80]. In human sperm, cAMP-dependent protein kinase (PKA) has been demonstrated to phosphorylate certain 20S proteasome subunits in vitro, thereby increasing chymotrypsin-like activity during the capacitation process [81]. Similarly, in mouse cardiac cells, PKA has been shown to regulate chymotrypsin-like activity and PGPH activity [43]. Our findings indicate that the proteasome subunits are phosphorylated by PKA during mouse spermatogenesis. A differential pattern of phosphorylated subunits was observed, with the highest intensity in the SS and DZ zones. Notably, this correlates with the assembly of the proteasome, as these same areas exhibited the largest quantity of the assembled 20S proteasome. These results are corroborated by reports in the literature from other cell models. In canine cardiomyocytes, exogenous or endogenous PKA stimulation rapidly enhances the assembly of the 26S proteasome and its enzymatic activity [82]. Notably, the effect of PKA activation on proteasome assembly and activity has been demonstrated to occur in vivo [82,83,84]. Furthermore, during spermatogenesis, the development of male germ cells is supported by the activation of FSH- and LH-stimulated PKA in Sertoli cells and in Leydig cells, respectively [85,86]. PKA subunits (RIα, RIIα, Cα1, and Cα2) are differentially expressed in mouse spermatogonia, spermatocytes, and spermatids, suggesting that PKA signaling may play a role in mitosis, meiosis, and/or spermiogenesis [85]. Consequently, PKA may also differentially regulate proteasome phosphorylation, and by extension, enzyme activity.

In conclusion, our results indicate that enzymatic activity, the level of 20S proteasome assembly, and its binding to regulatory complexes are differential in the stages of the seminiferous epithelium. This suggests the presence of proteasome subpopulations during spermatogenesis. This phenomenon may be attributed to differences in the cell types that comprise the various stages of the mouse seminiferous epithelium. Moreover, PKA may contribute to this differential activity through the phosphorylation of the proteasome.

## 4. Materials and Methods

### 4.1. Chemicals and Reagents

The following reagents were purchased from Sigma Chemical Co. (St. Louis, MO, USA): Nα-tosyl-L-lysine chloromethyl ketone hydrochloride (TLCK); Ponceau red; HEPES; ethylenediaminetetraacetic acid (EDTA); dimethyl sulfoxide (DMSO); sodium orthovanadate (Na_3_VO_4_); sodium fluoride (NaF); phenylmethylsulfonyl fluoride (PMSF); aprotinin; and leupeptin. The following compounds were purchased from Enzo Life Sciences (Farmingdale, NY, USA): N-succinyl-Leu-Leu-Val-Tyr-7-amino-4-methylcoumarin (Suc-LLVY-AMC); N-butoxycarbonyl-Gln-Ala-Arg-7-amino-4-methylcoumarin (Boc-QAR-AMC); Z-Leu-Leu-Leu-7-amino-4-methylcoumarin (Z-LLE-AMC); and N-Acetyl-N-methyl-l-isoleucyl-l-isoleucyl-N-[(1S)-3-methyl-1-[[(2R)-2-methyloxiranyl]carbonyl]butyl]-l-threoninamide (epoxomicin).

The antibodies used in this study, including primary and secondary antibodies, are listed in detail in Supplementary Table S1, which provides information on their names, manufacturers, catalog numbers, and dilution ratios. The chemiluminescence detection system and Immobilon P transfer membranes were purchased from Millipore Corporation (Bedford, MA, USA). The DC Protein Assay kit was purchased from Bio-Rad Laboratories Inc. (Hercules, CA, USA).

The deionized water used in these experiments was purified to 18 megohms with an EASY-pure UV/UF ion-exchange system (Barnstead/Thermolyne, Dubuque, IA, USA). All other chemicals were of analytical grade and obtained from standard sources.

### 4.2. Animals

Healthy and sexually mature male mice (*Mus musculus*) of strain CF-1 were acquired from the Animal Facility of our Faculty. The animals were maintained in a room under controlled conditions of temperature (22–25 °C) and a 12 h light/dark cycle, with ad libitum access to commercial pellets and tap water. For biological material collection, animals were euthanized by cervical dislocation. All euthanasia procedures were approved by the Ethics Committee on Scientific Research of the University of Antofagasta (CEIC-UA: N° Folio 454/2023).

### 4.3. Isolation and Dissection of Segments of the Seminiferous Tubules

Mice were euthanized via cervical dislocation. Following euthanasia, the testes were excised, decapsulated, and immediately transferred to a cooled phosphate-buffered saline (PBS) solution. The seminiferous tubules were mechanically separated from the interstitium using fine forceps under a transillumination dissection microscope [41]. The 12 stages of the mouse seminiferous epithelium cycle were grouped into four zones using the nomenclature described for this technique [40,41]. Briefly, four distinct portions were distinguished along the length of the tubule: the pale zone (PZ) contains stages IX–XI; the weak spot (WS) zone contains stages XII–I; the strong spot (SS) zone contains stages II–VI; and the dark zone (DZ) contains stages VII and VII (Figure 1). Once a segment was identified under transillumination, it was excised using ophthalmic scissors, causing increased internal pressure that expelled the luminal content. Subsequent washing in a homogenization buffer removed residual interstitial tissue but reduced luminal content and displaced free spermatozoa. Upon identification of the four zones, they were sectioned and stored at −20 °C for subsequent analysis, including the measurement of proteasomal enzyme activity, immunoprecipitation, SDS-PAGE, and immunoblotting.

### 4.4. Measurement of Proteasomal Enzyme Activity

To determine the different activities of the proteasome, 8–10 segments of the seminiferous tubule were cut for each study zone, which were then transferred to 1.5 mL Eppendorf tubes containing 80 µL of homogenization buffer (50 mM Hepes, 10% glycerol (*v*/*v*), 200 µM TLCK, pH 7.4). The segments were homogenized by sonication, which consisted of six repetitions of 30 sec at 60 W of power, using an ultrasonic sonicator (Virsonic, Gardiner, NY, USA). Subsequently, the sample was centrifuged at 16,000× *g* for 30 s in an IEC Micromax microcentrifuge to remove any remaining tissue. The supernatant was used for enzyme stock preparation. Proteasome enzymatic activity was determined for each protein extract using fluorogenic synthetic peptides specific to each activity. Briefly, 50 µL aliquots were incubated in a final volume of 995 µL of homogenization buffer for 15 min at 37 °C and 5% CO_2_, before 10 µM of substrate was added. The assay was run at 37 °C, and fluorescence was monitored with excitation at 380 nm and emission at 460 nm using a Shimadzu 5301 spectrofluorometer (Kyoto, Japan). The substrates used were as follows: Suc-Leu-Leu-Val-Tyr-AMC (Suc-LLVY-AMC) to measure chymotrypsin-like activity; butoxycarbonyl-Gln-Ala-Arg-AMC (Boc-QAR-AMC) to measure trypsin-like activity; and Z-Leu-Leu-Leu-Leu-AMC (Z-LLE-AMC) to measure PGPH activity. Proteasome activity was normalized by determining the total protein concentration of each extract using the DC Protein Assay (Bio-Rad, Richmond, CA, USA), following the manufacturer’s instructions. The resulting proteasome enzymatic activities are expressed in graphs as nmol AMC/mg protein/min, reflecting the specific activity of the proteasome. To verify that the substrates were specific to the three proteasome activities present in the protein extract, epoxomicin was used as a specific inhibitor of these activities, which irreversibly inhibited the activity of the catalytic subunits, inhibiting the three activities studied.

### 4.5. Immunoprecipitation

Proteasome immunoprecipitation was performed according to a previously described protocol [83] using an anti-PSMA7 proteasomal subunit antibody. Briefly, 400 μL of the sample containing 400 μg of protein was used for the immunoprecipitation assay. The protein concentration of each extract was determined and normalized using the DC Protein Assay (Bio-Rad, Richmond, CA, USA), following the manufacturer’s instructions. The sample was then incubated with 7 μL of anti-proteasome antibody immobilized on agarose at a concentration of 2 mg/mL. The reaction mixture was incubated overnight in an orbital shaker at 4 °C. Antigen-antibody–A/G-agarose protein complexes were concentrated at the bottom of the tube by centrifugation at 14,250× *g* for 30 s at 4 °C. The supernatants were discarded, and the pellets were washed twice with an immunoprecipitation (IPP) buffer (20 mM Tris HCl pH 7.5, 137 mM NaCl, 2 mM ethylenediaminetetraacetic acid (EDTA), 0.1% sodium dodecyl sulfate (SDS) (*w*/*v*), 0.5% C_24_H_39_O_4_Na (sodium deoxycholate, *w*/*v*), 1% Triton X-100 (*v*/*v*)), and once with PBS. The washed pellets were mixed with an SDS sample buffer (500 µM Tris-HCl, 10% SDS (*w*/*v*), 30% glycerol (*v*/*v*), 0.5% β-mercaptoethanol (*v*/*v*), and 0.5% bromophenol blue, pH 6.8 (*w*/*v*)) and heated in a boiling water bath for 5 min, and the supernatant was subjected to SDS-PAGE.

### 4.6. SDS-PAGE and Immunoblotting

Protein extracts from each study zone were obtained by homogenization by sonication in 90 µL of an RIPA buffer (50 mM Tris pH 7.4, 150 mM NaCl, 1% SDS (*w*/*v*), 1% NP-40 (*v*/*v*), 2 mM EDTA). Ten segments from each seminiferous tubule zone were used for each trial. The total extracted proteins were quantified using the DC Protein Assay (Bio-Rad, Richmond, CA, USA), following the manufacturer’s instructions. After quantification, the protein extracts were diluted in the SDS sample buffer and stored at −20 °C until use. The isolated proteins were separated by SDS-PAGE (60 mA, 120 min). Proteins were then transferred to polyvinylidene fluoride (PVDF) membranes at 250 mA for 60 min at 4 °C. The transfer was monitored using Ponceau red staining. Membranes were blocked in Tris-buffered saline with 0.1% Tween 20 (T-TBS, *v*/*v*) containing 5% nonfat dry milk (*w*/*v*) to block nonspecific binding during the immunoblotting process. They were then washed six times with T-TBS and incubated with the primary antibodies at 4 °C overnight. The membranes were then washed six times and incubated with the appropriate biotinylated secondary antibody for 1 h at room temperature. Next, the membranes were washed with T-TBS for the last time, and the blots were visualized by chemiluminescence (Amersham Corp., Sydney, Australia) according to the manufacturer’s instructions. Finally, the signal was imaged using the In Vivo F Pro molecular imaging system (Bruker Corporation, Billerica, MA, USA).

### 4.7. Stripping the PVDF Membranes

For the removal of antibodies from the membrane used in Western blotting, 15 mL of stripping buffer, consisting of 2% (*w*/*v*) SDS, 62.5 mM Tris, pH 6.7, and 100 mM β-mercaptoethanol (*v*/*v*), was added to the membrane for 1 h with constant shaking at 60 °C. The membrane was then washed six times for 10 min in TBS, blocked, and probed with an antibody against β-tubulin and the PSMA7 proteasome subunit (immunoprecipitation control).

### 4.8. Statistical Analyses

The results obtained in this study were statistically processed through an analysis of variance (ANOVA) followed by the Student–Newman–Keuls test for comparisons between zones. Comparisons between two individual zones were made using Student’s *t*-test. Densitometry analysis was performed using ImageJ 2.0 software and normalized with respect to the internal control tubulin. Values are presented as mean ± SEM values. In all cases, results with *p* < 0.05 were considered to be statistically significant.

## Data Availability

The data presented in this study are available on request from the corresponding author.

## Supplementary Materials

The following supporting information can be downloaded at https://www.mdpi.com/article/10.3390/ijms26020494/s1.
