# Supplementary material for: Differential Activity and Expression of Proteasome in Seminiferous Epithelium During Mouse Spermatogenesis
Source: Int J Mol Sci. 2025 Jan 9;26(2):494. doi: 10.3390/ijms26020494 (PMC11764840; doi:10.3390/ijms26020494)
Supplement: Supplementary file 1 [file ijms-26-00494-s001.zip › ijms-3375559-supplementary.pdf]

Supplementary Table S1. List of primary and secondary antibodies used in experiments.

Antibody Names	Manufacturer	Catalog Number	Dilution
Anti-phospho PKA substrates (RRXS*/T*)	Cell Signaling	9624	1:2000
Anti-α-4 (PSMA7)	Enzo Life Sciences	BML-PW8120	1:1000
Anti-β-4 (PSMB2)	Enzo Life Sciences	PSC-15-111	1:500
Anti-β-2 (PSMB7)	Enzo Life Sciences	BML-PW9300	1:1000
Anti-β-5 (PSMB5)	Enzo Life Sciences	BML-PW8895	1:1000
Proteasome 19S ATPase subunit Rpt6	Enzo Life Sciences	BML-PW9265	1:1000
Anti- PA200	Thermo Scientific	PA1-1961	1:500
Anti-β-tubulin (E7)	Developmental Studies Hybridoma Bank	AB_2315513	1:5000
HRP-Conjugated Goat Anti-Mouse IgG	Sigma-Aldrich	AP124	1:5000
HRP-Conjugated Goat Anti-Rabbit IgG	Sigma-Aldrich	AP307P	1:5000

* Corresponds to the phosphorylated forms of the amino acids serine and threonine.
